# Supplementary material for: Modified-Chronic Disease Score (M-CDS): Predicting the individual risk of death using drug prescriptions
Source: PLoS One. 2020 Oct 16;15(10):e0240899. doi: 10.1371/journal.pone.0240899 (PMC7567358; doi:10.1371/journal.pone.0240899)
Supplement: S1 Table — General criteria: at least 2 prescriptions in the observed year. For drugs which unambiguously identify the specific diagnosis (e.g., drug listed for patients with multiple sclerosis) we considered only 1 prescription. For drugs frequent also in minor and non-chronic conditions (e.g., proton pump inhibitors for peptic ulcer) we considered at least 3 prescriptions. *nonspecific association; #wrong association; §not refunded drug. (DOCX) [file pone.0240899.s001.docx]

**S1 Table. List of candidate conditions identified with their specific associated drugs and the minimum number of prescriptions (N). General criteria: at least 2 prescriptions in the observed year. For drugs which unambiguously identify the specific diagnosis (e.g., drug listed for patients with multiple sclerosis) we considered only 1 prescription. For drugs frequent also in minor and non-chronic conditions (e.g., proton pump inhibitors for peptic ulcer) we considered at least 3 prescriptions.**

| **Chronic Condition** | **Medication Class** | **N** | **Notes** |
| --- | --- | --- | --- |
| Cardiovascular disease | B01A: antithrombotic agents C01A: cardiac glycosides C01B: antiarrhythmics, class I and III C01D: vasodilators used in cardiac diseases C01E: other cardiac preparations C02: antihypertensives C07: beta blocking agents C08: calcium channel blockers C09: agents acting on the renin-angiotensin system | 2 | ADDED: prostaglandins (alprostadil) |
| Respiratory illness | R03: drugs for obstructive airways diseases | 3 | REMOVED: corticosteroids for systemic use*, mucolytics#, antiallergic agents§, other antiallergics§ |
| Exocrine pancreas failure | A09AA02: multienzymes (lipase, protease, etc.) | 3 | NEW CATEGORY: from cystic fibrosis |
| Cystic fibrosis | R05CB13: dornase alfa R07AX02-30: ivacaftor-ivacaftor and lumacaftor | 1 | EXTRACTED: exocrine pancreas failure* ADDED: ivacaftor and lumacaftor REMOVED: multienzymes* |
| Tuberculosis | J04A: drugs for treatment of tuberculosis | 3 |  |
| Cancer | H01CB: somatostatin and analogues [no somatostatin] L01: antineoplastic drugs [no nintedanib, hydroxycarbamide, mitoxantrone, cyclophosphamide] L02: endocrine therapy [no megestrol] L03AC: interleukins L03AX: other immunostimulants [no glatiramer] L04AX02-04-06: thalidomide, lenalidomide, pomalidomide V03AF: detoxifying agents for antineoplastic treatment | 1 | ADDED: thalidomide, lenalidomide, pomalidomide, detoxifying agents for antineoplastic treatment REMOVED: somatostatin#, betaxolol*, nintedanib*, hydroxycarbamide*, tretinoin*, rituximab*, mitoxantrone*, cyclophosphamide*, megestrol*, glatiramer#, opium alkaloids and derivatives*, levodropropizine*, drugs for neutropenia, emesis and nausea* |
| Acid related disorders/peptic ulcer | A02: drugs for acid related disorders | 3 |  |
| Chronic constipation | A06: drugs for constipation | 3 | NEW CATEGORY: from liver disease |
| Liver diseases | A05: liver therapy | 1 | EXTRACTED: chronic constipation* ADDED: liver therapy REMOVED CATEGORY: cirrhosis |
| Chronic hepatitis | L03AB10-11: peginterferon alpha 2b-2a J05AF08-10-11: adefovir dipivoxil, entecavir, telbivudine J05AP: antiviral for treatment of HCV infections | 2 | ADDED: specific reverse transcriptase inhibitors (J05AF) and antivirals for treatment for HCV infections REMOVED: interferon gamma#, interferon alpha 2a-2b*, interferon beta 1a-1b#, peginterferon beta 1a# |
| Diabetes | A10: drugs used in diabetes | 2 |  |
| Glaucoma | S01E: anti glaucoma preparations and miotics | 2 |  |
| Chronic renal disease | V03AE: drugs for treatment for hyperkalemia and hyperphosphatemia | 2 | REMOVED: other antianemic preparations* |
| Anaemias | B03: Antianemic preparations | 3 | REMOVED: colony stimulating factors |
| Bone diseases | A12A: calcium G03XC: selective estrogen receptor modulators H05: calcium homeostasis M05B: drugs affecting bone structure and mineralization | 3 | ADDE: basedoxifene |
| Infl.Bowel & rheumatologic | A07EA: corticosteroids acting locally A07EC: amino salicylic acid and similar agents L04AA13-18-24-26-33-37: leflunomide, everolimus, abatacept, belimumab, vedolizumab, baricitinib L04AB: tutor necrosis factor alpha inhibitors L04AC03-07-08-10-14: anakinra, tocilizumab, canakinumab, secukinumab, sarilumab P01BA02: hydroxychloroquine | 2 | ADDED: canakinumab, secukinumab, sarilumab REMOVED: antithymocyte immunoglobulin (rabbit)#, mycophenolic acid*, sirolimus*, natalizumab#, eculizumab#, fingolimod#, tofacitinib*, teriflunomide#, apremilast#, alemtuzumab*, ocrelizumab#, antiinflammatory and antirheumatic drugs*, topical products for joint and muscular pain§, chloroquine* |
| Pain and inflammation | M01A: anti-inflammatory and antirheumatic products, non-steroids N02: analgesic drug N03AX12-16: gabapentin, pregabalin N06AX21: duloxetine | 3 | ADDED: gabapentin, pregabalin, duloxetine |
| Hyperuricemia/gout | M04A: antigout preparations | 2 |  |
| Dermatological severe | D05: anti-psoriatic drugs L04AA32-C05-13-16: apremilast, ustekinumab, ixekizumab, guselkumab D10: acne drugs D11AH: agents for dermatitis, excluding corticosteroids D07: corticoids, dermatological preparations | 2 | NEW CATEGORY: from psoriasis and acne ADDED: specific selective immunosuppressants (L04A), antiacne preparations for systemic use, tracrolimus, pimecrolimus, alitretinoin, dupilumab, corticosteroids dermatological preparations REMOVED: antiacne preparations for topical use |
| Transplantation | L04AA04-06-10-18-C02-D02: antithymocyte immunoglobulin (rabbit), mycophenolic acid, sirolimus, everolimus, basiliximab, tacrolimus | 2 | ADDED: immunosuppressant drugs (L04A) REMOVED: ciclosporin*, azathioprine* |
| Hyperlipidaemia | C10: lipid modifying agents | 2 |  |
| HIV | J05AD: phosphonic acid derivatives J05AE: protease inhibitors J05AF01-02-04-05-06-07-09: zidovudine, didanosine, stavudine, lamivudine, abacavir, tenofovir disoproxil, emtricitabine J05AG: non-nucleoside reverse transcriptase inhibitors J05AR: antivirals for treatment of HIV infections, combinations J05AX: other antivirals | 1 | ADDED: other antivirals, antivirals for treatment of HIV infections (J05AR) REMOVED: nucleosides and nucleotides excluded reverse transcriptase inhibitors*, adefovir dipivoxil#, entecavir#, telbivudine#, clarithromycin*, rifabutin*, atovaquone*, pentamide isethionate* |
| Hypothyroidism | H03A: thyroid preparations | 2 | REMOVED: thiamazole# |
| Epilepsy | N03A: antiepileptic drugs (no phenobarbital, clonazepam, carbamazepine, valpromide, lamotrigine, gabapentin, pregabalin, retigabine N05CD08: midazolam | 2 | REMOVED: phenobarbital*, clonazepam*, carbamazepine*, valpromide*, lamotrigine*, gabapentin*, pregabalin*, retigabine* |
| Dementia | N06D: dementia drugs N06BX13: idebenone | 2 | ADDED: idebenone |
| Parkinson’s disease | N04: antiparkinsonism drugs [no bromocriptine] | 2 | REMOVED: bromocriptine* |
| Depression, anxiety, OCD | N05B: anxiolytic drugs N06A: antidepressant drugs [no duloxetine] | 2 | REMOVED: duloxetine*, midazolam#, idebenone#, monoamine oxidase B inhibitors#, fluoxetine and psycholeptics* |
| Bipolar disorders | N05AN: lithium | 2 |  |
| Psychosis | N05A: antipsychotic drugs [no lithium] | 2 | REMOVED: lithium# |
| Multiple sclerosis | L03AB07-08-13: interferon beta 1a-1b, peginterferon beta 1a L03AX13: glatiramer L04AA23-27-31: natalizumab, fingolimod, teriflunomide L04AX07: dimethyl fumarate | 1 | NEW CATEGORY |
| Haemorrhagic diathesis | B02: antihemorrhagic | 2 | NEW CATEGORY |
| Allergic disorders | R06: antihistamines for systemic use | 3 | NEW CATEGORY |
| Addictive disorders | N07B: drugs used in addictive disorders | 2 | NEW CATEGORY |

Legend: *nonspecific association; #wrong association; §not refunded drug.
